# Metagenomic Chromosome Conformation Capture (3C): techniques, applications, and challenges

**DOI:** 10.12688/f1000research.7281.1

**Published:** 2015-11-30

**Authors:** Michael Liu, Aaron Darling

**Affiliations:** 1 ithree institute, University of Technology Sydney, Sydney, NSW 2007, Australia

**Keywords:** Hi-C, 3C, metagenomics

## Abstract

We review currently available technologies for deconvoluting metagenomic data into individual genomes that represent populations, strains, or genotypes present in the community. An evaluation of chromosome conformation capture (3C) and related techniques in the context of metagenomics is presented, using mock microbial communities as a reference. We provide the first independent reproduction of the metagenomic 3C technique described last year, propose some simple improvements to that protocol, and compare the quality of the data with that provided by the more complex Hi-C protocol.

## Introduction

Metagenomics has been proposed as a means to characterize the microbial communities that are pervasive in our environment ( Handelsman, 2004). Current metagenomic protocols, however, fail to capture critical information on the organisation of genetic material in microbial communities, as the fine-scale structure of the community and linkage among DNA sequences is intentionally destroyed by cell lysis and DNA shearing steps prior to sequencing. Computational methods of sequence binning attempt to assign sequences to the species or strains that were present in the sample, thereby inferring the linkage information destroyed by sample processing, but these methods have limited resolution despite many years of development ( Lindgreen *et al.*, 2015; Peabody *et al.*, 2015).

Chromosome conformation capture (3C) and related approaches offer an alternative strategy that allows the spatial organization of genetic material in a microbial community to be preserved and measured, either via high throughput sequencing or other assays. In 3C, the fine-scale structure of the sample is preserved via reversible crosslinking, typically by soaking the sample in formaldehyde immediately after collection ( Dekker *et al.*, 2002). The sample is then subjected to cell lysis and further steps are applied to interrogate the spatial structure in the sample.

Published protocols for coupling 3C with metagenomics involve restriction digestion, followed by a *proximity ligation*, followed by crosslink reversal, DNA collection, optional enrichment for ligation junctions, and sequencing library preparation ( Beitel *et al.*, 2014; Burton *et al.*, 2014; Marbouty *et al.*, 2014). The proximity ligation is a key step wherein a DNA ligation reaction is carried out under highly dilute conditions. The low concentration of sample material favors ligation events among DNA strands which are crosslinked together in the same molecular complex. Crucially, this allows separate DNA macromolecules, e.g. a chromosome and a plasmid, or two chromosomes that were co-bound in a protein complex, to become ligated to each other ( Beitel *et al.*, 2014). These ligation junctions can then be identified via high throughput sequencing. The rate at which such ligation events are observed in the data is highly correlated with the frequency at which the DNA was in close physical contact at the time of sample crosslinking ( Lieberman-Aiden *et al.*, 2009).

Several other methods can support direct measurement or inference of linkage among metagenomic DNA sequences. We describe these below. Metagenomic 3C has several advantages relative to these other methods, along with some disadvantages.

### Single cell sequencing

Single cell sequencing methods can capture data on a relatively large fraction of the genetic material in a cell (10–80% depending on the whole genome amplification conditions). However single cell techniques are vulnerable to reagent and equipment contamination and depend on cells being readily separable, making them difficult to deploy widely. Moreover, single cell techniques gather data on only a small fraction of the cells in a sample rather than the entire population.

### Long read single molecule sequencing

The Pacific Biosciences and Oxford Nanopore platforms implement sequencing technologies that can read DNA strands up to 100 kilobases ( Laver *et al.*, 2015) and possibly more. Long sequence reads capture more information about the arrangement of genes into chromosomes than is available in short (<1000nt) reads typical of other sequencing technologies. Single molecule sequence reads currently have accuracy ranging from 80–90%, which is sufficient for detecting genes but offers only limited ability to identify single nucleotide variants and indels ( Quick *et al.*, 2014). Consensus signal approaches such as Circular Consensus Sequencing can help to overcome the error in single molecule sequencing but do so at the expense of read length or throughput ( Larsen *et al.*, 2014). These methods read single molecules and therefore they are unable to identify relationships between plasmids and host chromosomes without being coupled to a library preparation method like 3C or Hi-C.

### Correlated coverage binning

This strategy leverages the observation that genetic material present in the same species or strain changes in abundance over time & space in a highly correlated manner. By generating metagenomic data on an environment across multiple time points, sampling sites, or even different cell lysis treatments, it becomes possible to reconstruct linkage information by identifying sequences whose abundances are highly correlated across samples ( Albertsen *et al.*, 2013; Alneberg *et al.*, 2014; Imelfort *et al.*, 2014). The power to detect such associations grows with the number of samples and the extent of change across samples ( Alneberg *et al.*, 2014). This approach has the advantage of being relatively simple to implement, only requiring the additional effort to collect and process a larger number of samples. A potential drawback is that in recombining populations, the abundance of a particular gene, plasmid, or polymorphism may not correlate strongly with one particular host species' abundance, leading to a failure to correctly identify the linkage relationship. Plasmids and bacteriophage may have copy number dynamics that are independent of host chromosomes, potentially making some associations difficult to detect. Finally, this approach does not provide direct information to order & orient assembly contigs into genome-scale scaffolds, however the inferred linkage information could in principle be used to eliminate ambiguity in assembly graphs and so yield more contiguous assemblies.

## Metagenomic 3C

Metagenomic 3C has thus far been implemented in two protocols. Text box 1 gives an overview of these protocols and Table 1 highlights the main differences in the quality of data generated by each protocol. The Hi-C approach was the first to be described in the context of metagenomics ( Beitel *et al.*, 2014; Burton *et al.*, 2014), and involves steps that enrich the sample for proximity ligations. The basic metagenomic 3C approach has the advantage of being simpler to execute in the laboratory ( Marbouty *et al.*, 2014).

We have succeeded in implementing and extending the protocol first described by Marbouty *et al.*, 2014 on a mock community to facilitate a detailed comparison of metagenomic 3C and Hi-C. Our extension of the original protocol adds a bead purification step following crosslink reversal and replaces the shearing & adapter ligation for sequencer library preparation with a tagmentation reaction. This in turn reduces input material requirements by several fold, enabling the reactions to be scaled down and reducing reagent cost. The details of the extended protocol and accession numbers for the associated data sets can be found in the Supplementary material.

Several challenges emerge in applying 3C protocols to microbial communities. Samples often consist of heterogeneous cell types. The thick walls of some cells may affect the extent of crosslinking, causing some cells to crosslink more extensively than others. High formalin concentrations lead to reduced DNA recovery in later stages of the protocol. Data from experiments using a range of formalin concentrations on the same sample suggest that concentrations between 2 and 3% provide an optimal trade-off between proximity ligation rate in gram positive cells and DNA yield (see Supplementary material). However, these data reflect only a small number of species relative to the currently described microbial diversity.

Microbial communities can consist of organisms with a wide range of genomic G+C composition, and this must be considered when selecting a restriction enzyme to use in 3C and related protocols. Data on synthetic communities shows that density of restriction sites is directly proportional to the rate of observed proximity ligation events in metagenomic 3C data. For example, a library created using the enzyme HpaII (recognition site C^CGG) yields very few reads with proximity ligation junctions for *S. aureus* (32% G+C) but for *P. aeruginosa* (67% G+C) up to 6.5% of reads contain proximity ligation junctions. Therefore it may be advantageous to process samples in parallel with two or more enzymes having diverse recognition sites.

**Text box 1.**
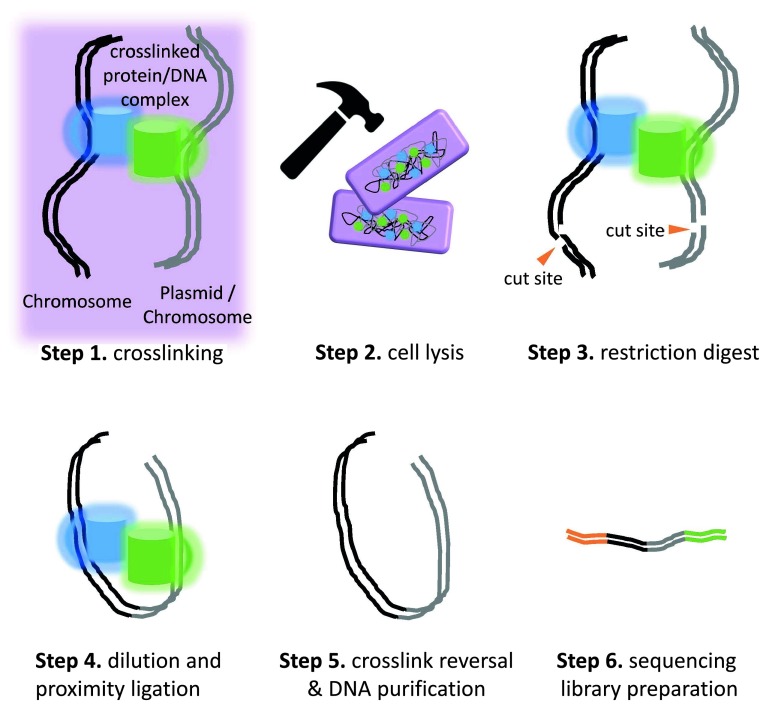
3C and proximity ligation methods. Chromosome conformation capture (3C) was first developed as a means to determine the average three dimensional chromosome structure in a population of cells, for a single species ( Dekker *et al.*, 2002). This general approach was later coupled with high throughput DNA sequencing ( Lieberman-Aiden *et al.*, 2009), providing a means to generate detailed 3D structure models of chromosomes. Many extensions of the 3C technique have been developed ( Dekker *et al.*, 2013). The basic 3C protocol involves an initial step of reversible crosslinking, typically via formaldehyde at 1–3%. This step crosslinks proteins to each other and to DNA. The formaldehyde is then quenched and the cells are lysed either enzymatically or via physical disruption. Next, a restriction digestion is carried out using a 4- or 6-cutter that leaves a single-stranded overhang. Subsequently the sample is placed in a large volume DNA ligase reaction; yielding conditions that strongly favor the ligation of free ends that are co-bound in a protein complex. This step is referred to as *proximity ligation*. After proximity ligation, the crosslinks are reversed via heat incubation and the DNA is purified via proteinase K & RNAse digestion and EtOH precipitation. Finally, the purified DNA is ready for standard high throughput sequencing library preparation, for example via adapter ligation and enrichment PCR. Hi-C extends the protocol described above by incorporating steps that enrich the final sequencing library for proximity ligation events. In Hi-C, the single stranded overhangs left after the restriction digest are filled with biotinylated nucleotides. The proximity ligation which follows is thus a blunt-end ligation and the junctions contain biotinylated nucleotides. Biotinylated nucleotides must be removed from any remaining unligated free ends. In the final steps of sequencing library preparation, fragments containing the biotinylated ligation junctions can be captured on streptavidin-coated magnetic beads, yielding a library substantially enriched for proximity ligations ( Lieberman-Aiden *et al.*, 2009).

**Table 1.** Comparison of 3C and Hi-C for metagenomics.

	3C	Hi-C
Proximity ligation read rate	Up to 6.5%	4% ( Beitel *et al.*, 2014) or 12–51% ( Burton *et al.*, 2014)
Resolution limit	1–2kbp	1–2kbp (4 cutter) or 15–30kbp (6 cutter)
Marked ligation junctions	No	Yes
Difficulty of library prep	hard	very hard
Erroneous association rate	<1%	<1%
Requires separate metagenomic library	No	Yes

Table 1. Differences in the features of metagenomic 3C and Hi-C are listed. The proximity ligation read rate indicates the fraction of all reads that contain proximity ligation events. For Hi-C the rate varies widely in published data. The resolution limit is dictated by the density of restriction cut sites in the chromosome, which are typically more dense when using a 4-cutter (3C or Hi-C), than with a 6-cutter (Hi-C only). Marked ligation junctions are created as a by-product of the end-filling in Hi-C and can be identified as a tandem duplication of the overhang sequence in the data. The erroneous association rate is defined as the fraction of read pairs found to associate two different species or strains in mock community experiments.

## Applications of metagenomic 3C

### Reconstructing genomes from metagenomes

The data produced by metagenomic 3C or Hi-C can be used to address a range of questions in microbial community analysis. Chief among these is reconstruction of the so-called population genomes of each species present in a microbial community. A population genome does not reflect the genome of an individual cell in the community, but rather is a consensus genome sequence describing the genetic material present in a collection of closely related cells, e.g. a population or species. The population genome may represent an amalgamation of many closely related strains each with their own strain-specific gene content and mutations. The extent of such microdiversity among strains has a strong influence on the ability of current sequence assembly algorithms to reconstruct a metagenomic assembly. Once recovered, the population genomes can support a range of downstream analysis such as metabolic network reconstruction for individual community members. Predicted metabolic networks can in turn be used to inform analysis of species interactions and help guide strategies for identifying and cultivating microbes of interest ( Imelfort *et al.*, 2014; Parks *et al.*, 2015).

Current approaches for reconstructing population genomes are relatively simplistic and involve a first step of mapping the 3C read pairs to the metagenomic assembly, counting the number of links found among each contig in the read pair data, and then using a clustering algorithm to group contigs by population/species. Several clustering algorithms have been explored for this task. Beitel *et al.*, 2014 applied Markov clustering and found that use of a low inflation parameter in the algorithm led to clusters that accurately reflect population genomes. Marbouty *et al.*, 2014 used Louvain clustering and were able to achieve similarly accurate results on simple test communities. Both of these algorithms have the advantage that prior knowledge of the number of population genomes is not required. Burton *et al.*, 2014 applied a custom algorithm that requires the number of population genomes in the sample to be known *a priori*. This requirement is likely to pose a difficulty in cases where independent lines of evidence are unable to yield a reliable estimate of the number of population genomes in a sample.

**Figure 1.**
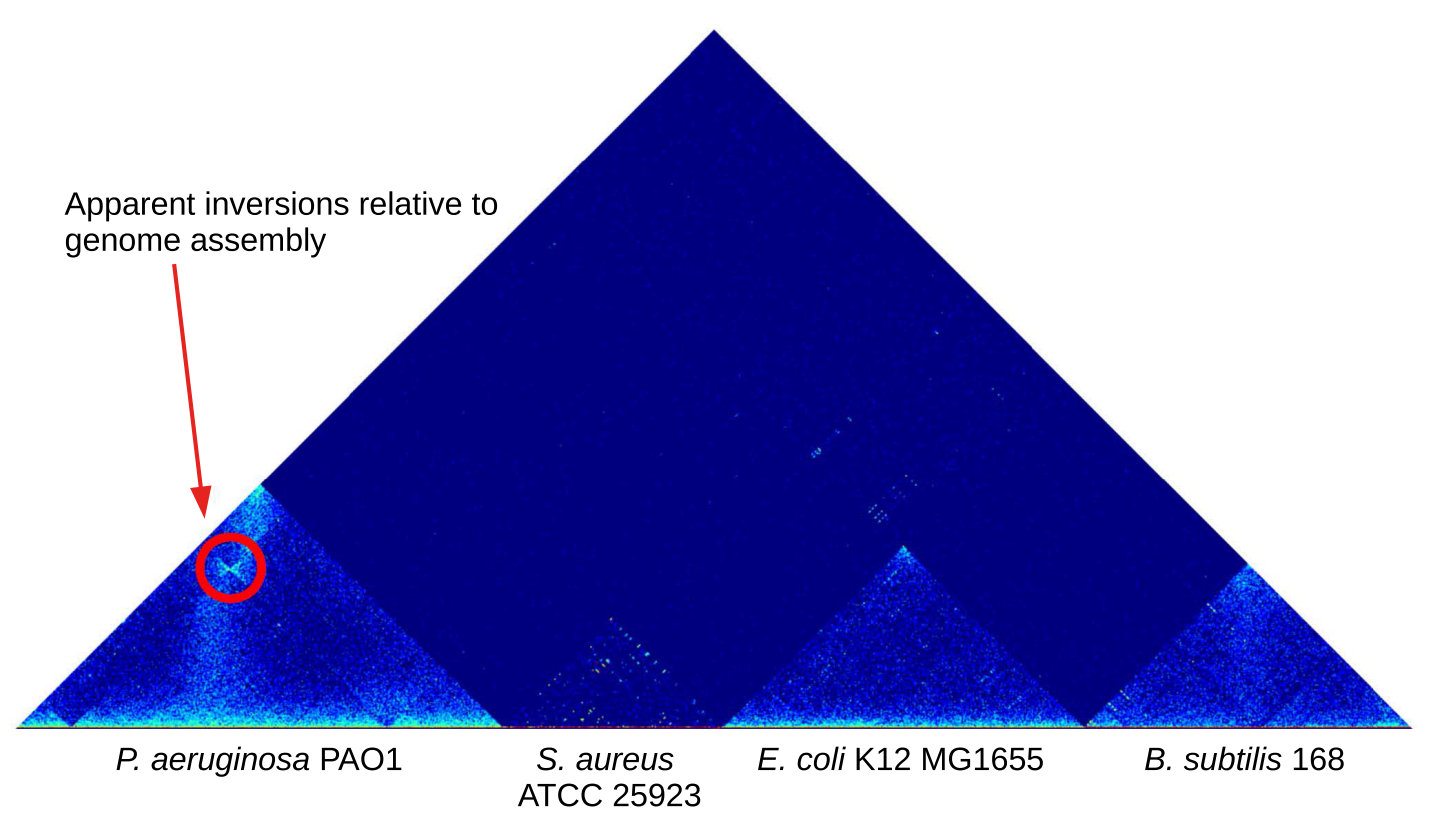
3C/Hi-C heatmap. Contact map of chromatin interactions identified by metagenomic 3C. A synthetic community of four bacterial isolates was subjected to metagenomic 3C and the resulting read data mapped back to reference chromosome assemblies. Heat intensity is proportional to the number of read pairs associating the two chromosome regions. In *P. aeruginosa* and *B. subtilis*, the two arms of the circular chromosome are colocalized, as reflected in the column of intense heat emanating from the middle of their chromosomes. Erroneous cross-species associations are seen to be rare (deep blue field).

In addition to its use in reconstructing population genome content, metagenomic 3C can in principle be used to guide the scaffolding of metagenomic assembly contigs. Hi-C data has already been demonstrated to facilitate chromosome-scale scaffolding of large eukaryotic genomes ( Burton *et al.*, 2013; Marie-Nelly *et al.*, 2014). When scaffolding microbial genomes, the much greater resolution afforded by 4-cutters (as used in the basic metagenomic 3C protocol) is likely to be essential for accurately ordering & orienting contigs in population genomes. The signal available for scaffolding can be visualized using the contact map concept, as shown in Figure 1. When the contigs are correctly ordered and oriented the majority of contacts occur locally, obeying a distance-decay relationship dictated by polymer physics ( Marie-Nelly *et al.*, 2014). Figure 1 highlights an exception to this, where the strain used in the laboratory has undergone rearrangement relative to the finished reference genome.

### Tracking plasmids, bacteriophage, and mobile DNA

Metagenomic 3C offers the exciting possibility to quantify the frequency of association between mobile DNA such as plasmids and host chromosomes. In the simplest scenario, such data could be used in a purely descriptive capacity, to document the relationships between plasmids and hosts in various microbial ecosystems. Another possibility would be to characterise how the relationships between host chromosomes and plasmids change over time in response to external stimuli, for example antibiotic exposure. 3C-based protocols that employ 4-cutter enzymes are likely to be essential for such applications, since the use of a 4-cutter increases the likelihood that suitable cut sites will exist in small plasmids.

In principle a similar strategy could be applied to characterise relationships between host chromosomes and bacteriophage or other types of mobile DNA. Previous work in mouse models has suggested that bacteriophage in the mouse gut selectively transduce antibiotic resistance genes and broaden their host range in response to antibiotic treatment ( Modi *et al.*, 2013). Application of metagenomic 3C techniques in this context remains unexplored, although current protocols and computational techniques are adequate to support such applications.

## Future directions

Metagenomic 3C provides information on the spatial organisation of genetic material in microbial communities. This type of information is valuable and highly complementary to data generated by other strategies and technologies. In particular, the ability to link separate DNA polymers which are localized in the same cell creates opportunities for study that would be intractable with classic shotgun sequencing strategies, whether using long reads or not.

Several barriers currently prevent ready application of metagenomic 3C and related methods to microbial communities. Naturally occurring microbial communities can harbour a milieu of live and dead cells, along with free DNA and protein. At the time of this writing, no application of the technique has yet been reported for a natural environmental sample. Marbouty *et al.*, 2014 described an application to a sample sourced from Seine river sediments, however, that sample was subjected to an enrichment culture prior to formalin fixation. The enrichment culture presumably created a population of intact cells and reduced the prevalence of free DNA in the sample.

Classic 3C and Hi-C protocols require large amounts of sample material, but microbial communities of interest can be of very limited biomass, for example subgingival dental plaques or individual soil particles. Improving the efficiency of the metagenomic 3C protocol will be essential before it can be applied to such sample types. Several possible avenues exist to improve the reaction efficiency, elements of which have already been described in the context of single-cell Hi-C experiments on mammalian cells ( Nagano *et al.*, 2013).

A further major barrier to analysis of metagenomic 3C data is the presence of strain-level microdiversity in a sample. The existence of even just two strains with genomes around 98% average nucleotide identity is sufficient to cause extensive fragmentation in genome assemblies, depending on the assembly algorithm. The resulting assembly contigs can be too small to harbor restriction sites and therefore will fail to cluster into population genomes. In principle, advanced computational methods which operate directly on genome assembly string graphs ( Myers, 2005) instead of their contig-based representations could solve this problem. However, such computational tools do not currently exist for metagenomic 3C data analysis. It is worth noting that this problem also impacts the use of other strategies for generating population genomes such as correlated coverage binning.

Hi-C data has been demonstrated to facilitate phasing human chromosomes ( Selvaraj *et al.*, 2013), and Beitel *et al.*, 2014 showed that metagenomic Hi-C data had characteristics that would support resolution of the genotypes of two *E. coli* strains in a synthetic mixture. Much work remains before 3C or Hi-C could actually be applied to strain resolution, however. The number of genotypes present in a microbial community is unknown *a priori*, and the degree of divergence among genotypes is also unknown but has a major influence on the technique’s resolving power. Substantial investment will be required to develop tools for statistical inference on the genotypes present in samples characterized by metagenomic 3C sequencing. The fact that the number of genotypes and their divergences are unknown *a priori* will add significant complexity to the algorithms. It is likely the case that reconstructing the genotypes of individual cells in the sample will remain impossible, but inference algorithms may instead compute a probability distribution over cellular genotypes. Such a probability distribution could support testing & rejection of specific hypotheses, for example whether gene A and B are subject to an epistatic interaction, or whether population X is significantly more diverse than population Y. In the extreme case where strain genotypes are separated by just two variant sites in distant chromosomal locations, a very large amount of 3C data would be required to generate enough read pairs covering the two sites to estimate their frequency of linkage. This is due to nature of 3C data, and reflects the fact that distantly located sites rarely interact in the cell in most cases ( Beitel *et al.*, 2014; Marie-Nelly *et al.*, 2014). This represents a fundamental limitation of metagenomic 3C and highlights a need for complementary strategies such as the single cell or correlated coverage techniques.

## Supplementary materials

### Supplementary Figure S1

Raw, unnormalized rate of proximity ligation products in metagenomic 3C libraries, as a function of formalin concentration. A synthetic microbial community was subjected to metagenomic 3C library prep & sequencing at a range of formalin concentrations, and the fraction of read pairs mapping at distances >1000nt was taken as an estimate of the proximity ligation read rate.

### Supplementary information

Protocol for the construction of metagenomic 3C (Meta3C) libraries, Illumina sequencing, analysis of metagenomic 3C data and analysis of the Burton *et al.*, Hi-C 2014 data.
