# Supplementary material for: Metagenomic Chromosome Conformation Capture (3C): techniques, applications, and challenges
Source: F1000Res. 2015 Nov 30;4:1377. [Version 1] doi: 10.12688/f1000research.7281.1 (PMC4798154; doi:10.12688/f1000research.7281.1)
Supplement: Supplementary file 1 [file f1000research-4-7846-s0000.tgz › d371679f-e5f5-4387-ab63-6a380448aac8.docx]

Supplementary information

**Construction of metagenomic 3C (Meta3C) libraries**

The sequence read data associated with the Marbouty et al 2014 metagenomic 3C protocol is not publicly available. Therefore we replicated the procedure to create a publicly available dataset for comparison with metagenomic Hi-C.

We modified the metagenomic 3C protocol of Marbouty et al 2014 to improve reaction efficiency. The protocol is as follows. Laboratory strains of *Bacillus subtilis* SU5, *Escherichia coli* K12 MG1655, *Staphylococcus aureus* ATCC25923 and *Pseudomonas aeruginosa* PAO1 were used for the construction of the meta3C library. For each strain, cells were grown in defined rich Luria-Bertani (LB) medium at 37°C. Cells from the different species were then mixed and aliquoted into four tubes where they were cross-linked with fresh formaldehyde (Amresco, USA) at 2%, 3%, 4%, and 5% final concentrations in each tube for up to an hour at room temperature (RT) followed by 60 minutes at 4°C. Formaldehyde was quenched with a final concentration of 0.25M glycine for 10 minutes at RT followed by 15 minutes at 4°C. Fixed cells were collected by centrifugation, frozen on dry ice and stored at -80°C until use. A frozen pellet of approximately 1 x 10^10^ cells was thawed on ice and resuspended in a final volume of 500uL 1X TE buffer (pH 8.0, Ambion) before adding 4uL of lysozyme mix Ready-lyse (Epicentre, Madison, WI, USA) followed by incubation at RT for 20 minutes. The cells were further lysed using *lysing matrix B tubes* (MPBio) in a FastPrep instrument (6500rpm, 2X30 seconds, MPBio). The lysate was then transferred into a new tube and SDS was added to a final concentration of 0.5% followed by 10 minutes RT incubation. Two 50uL aliquots of lysed cells were put into tubes containing 450uL of digestion mix (1× NEB CutSmart buffer [New England Biolabs], 1% Triton X-100, and 100U HpaII enzyme [NEB; C^CGG]). The chromatin was digested for 3 hours at 37°C and pooled into one tube followed by dilution with 8mL ligation buffer (1 X ligation buffer NEB without ATP, 1mM ATP, 0.1mg/mL BSA, 2000 units (cohesive end units, CEU) of T4 DNA ligase [New England Biolabs]). Note: the standard protocol uses 125 Weiss units = 25000 CEU for this step, but we found 2000 CEU to be sufficient on these mock microbial communities. The low concentration may not work on natural samples which can contain enzyme inhibitors. Ligation was performed at 16°C for 4 hours followed by a reverse cross-linking step consisting of an overnight (ON) incubation at 65°C with 250ug/mL proteinase K in EDTA (Promega). DNA containing proximity ligated chromatin junctions was precipitated with 900uL of 3M sodium acetate (pH 6.0) and 9mL of isopropanol (Sigma-Aldrich) with a one hour incubation at -80°C. DNA was pelleted by centrifugation and resuspended in 500uL of 1X TE buffer (pH 8.0) followed by RNA removal with a final concentration of 3mg/mL RNAse A (Invitrogen) for one hour at 37°C. The DNA was then cleaned with SPRIselect beads (1.8X ratio, Beckman Coulter) and eluted with 20uL of UltraPure water (Invitrogen). The DNA was then quantified with both the Agilent 2100 Bioanalyzer using High Sensitivity DNA analysis kit (Aglient Technologies) and the Qubit HS DNA kit on a Qubit fluorimeter (Invitrogen).

**Illumina sequencing**

An aliquot of 2ng of 3C library was used as input to the Illumina Nextera XT library preparation protocol. Tagmentation of genomic DNA and PCR amplification of tagged DNA were performed following the manufacturer’s instructions, using different barcodes to identify samples that were fixed at 2, 3, 4, and 5% formalin concentrations. DNA fragments ranging in size from 400-600 bp were purified using SPRISelect beads (Beckman Coulter). The four samples were pooled and sequenced with Illumina MiSeq V2 chemistry, generating paired-end 29nt reads. Between 1.8M and 6.7M read pairs were generated for each sample. The reads were deposited to the NCBI Short Read Archive, accession numbers SRX1116284, SRX1117095, SRX1117096, and SRX1117098.

**Analysis of metagenomic 3C data**

Reads from each sample were mapped to reference genome assemblies (NCBI accessions NC_002516.2, CP009361.1, CP009362.1, NC_000913.3, NC_000964.3) using bowtie2 version 2.2.5 in paired-end mode with default parameters. The mapped reads were then analysed to calculate rates of proximity ligation products in each metagenomic 3C library as a function of the formalin concentration used for crosslinking. An estimate of the proximity ligation product rate was estimated as the fraction of read pairs in the library mapping >= 1000nt apart on the same chromosome relative to all read pairs mapping to that chromosome, with an estimated mapping quality >= 20 (MAPQ >= 20). Proximity ligation rate estimates are given in Figure S1. Python & R scripts implementing these analyses are available from http://github.com/koadman/3Creview

The highest observed proximity ligation read rate was 6.5%, for *P. aeruginosa* when fixed with 2% formalin. The proximity ligation rate for *P. aeruginosa* decreases across the range of formalin fixation conditions to a low of about 2%, whereas for other organisms the rate increases over this same range. The optimal metagenomic 3C formalin concentration for this group of gram positive & negative organisms appears to be around 3%. We observed very few proximity ligation events for *S. aureus,* which we attribute to the fact that *S. aureus* has a low G+C genome and therefore harbors few HpaII cut sites (C^CGG). The greater distance between cut sites permits only longer distance chromatin interactions to be captured, and these occur at much lower rates. Of note, a small number of read pairs were observed to link the *S. aureus* plasmid to another chromosome. Of these, an estimated 95%, 91%, 75%, and 50% link the plasmid to the *S. aureus* main chromosome in the 2%, 3%, 4%, and 5% fixation conditions, respectively.

**Analysis of Burton et al 2014 Hi-C data**

The Hi-C data associated with Burton et al 2014 was obtained from SRA (accession SRR1263047) and mapped to a selection of reference genomes of bacteria in the sample (accessions NC_000964.3, NC_004129.6, NC_005296.1, NC_005297.1, NC_005791.1, NC_006840.2, NC_006841.2, NC_006842.1, NC_007650.1, NC_007651.1, NC_009441.1) as described above. Analysis of the per-organism proximity ligation read rates was carried out as above. Proximity ligation read rates range from an estimated 12% for *Burkholderia thailandensis* chromosome II to 51% for *Flavobacterium johnsoniae*.


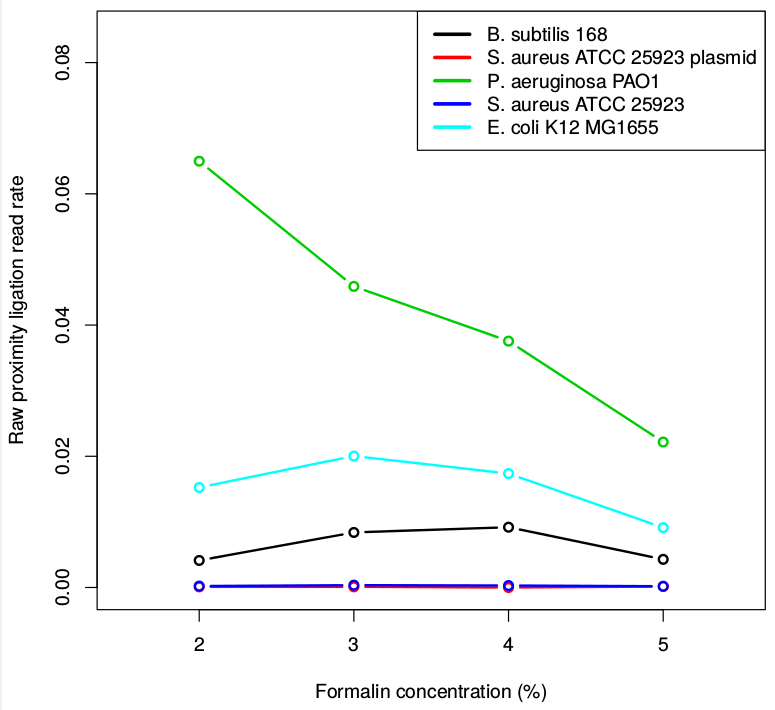


**Figure S1.** Raw, unnormalized rate of proximity ligation products in metagenomic 3C libraries, as a function of formalin concentration. A synthetic microbial community was subjected to metagenomic 3C library prep & sequencing at a range of formalin concentrations, and the fraction of read pairs mapping at distances >1000nt was taken as an estimate of the proximity ligation read rate.
