# Supplementary material for: Metagenomic Chromosome Conformation Capture (3C): techniques, applications, and challenges
Source: F1000Res. 2015 Nov 30;4:1377. [Version 1] doi: 10.12688/f1000research.7281.1 (PMC4798154; doi:10.12688/f1000research.7281.1)
Supplement: Peer review history

# Peer review: Referee response for version 1

**DOI:** 10.5256/f1000research.7846.r11377

**Published:** 2016-03-15

**Authors:** Titus Brown

**Affiliations:** 1 Department of Population Health and Reproduction, University of California Davis, Davis, CA, USA

This is a thorough discussion of using 3C for metagenome reconstruction. I was a little surprised by the elaborate review-style article together with some experimental data but I think it works well.

I agree with the points made by the other reviewer (Mick Watson). It would be nice to see more complex metagenomes tackled!

One final suggestion - the reference to graph genomes is poor and rather insufficient - there's been a lot of recent work in this area. I refer you to Dilthey *et al,* 2015, Improved genome inference in the MHC using a population reference graph for one reference.

I have read this submission. I believe that I have an appropriate level of expertise to confirm that it is of an acceptable scientific standard.

## References

[1] DiltheyACoxCIqbalZNelsonMRMcVeanG: Improved genome inference in the MHC using a population reference graph. *Nat Genet*.2015;47(6) : 10.1038/ng.3257 682-8 10.1038/ng.3257 25915597PMC4449272

# Peer review: Referee response for version 1

**DOI:** 10.5256/f1000research.7846.r11375

**Published:** 2016-01-25

**Authors:** Mick Watson

**Affiliations:** 1 The Roslin Institute, University of Edinburgh, Edinburgh, UK

Liu and Darling present an excellent review of the application of 3C capture techniques to metagenomic data analysis and I recommend indexing.

There are two issues I would have liked to have seen discussed/presented more:

1. Often metagenomic samples undergo bead bashing in order to disrupt the gram+ cell walls, and this often results in highly fragmented DNA.  It would be interesting to hear the thoughts of the authors on how this might affect the results from 3C capture techniques.
2. Figure 1 shows a beautiful reconstruction of 4 genomes.  However, many real environmental samples contain 1000+ genomes.  The authors discuss this in great detail, and the problems that ensue; however, synthetic metagenomes exist consisting of more than 4 but less than 1000, and I wonder why nobody has applied 3C techniques to those synthetic communities?

I have read this submission. I believe that I have an appropriate level of expertise to confirm that it is of an acceptable scientific standard.
